# Vascular and Hepatic Impact of Short-Term Intermittent Hypoxia in a Mouse Model of Metabolic Syndrome

**DOI:** 10.1371/journal.pone.0124637

**Published:** 2015-05-18

**Authors:** Wojciech Trzepizur, Abderahim Gaceb, Claire Arnaud, Christophe Ribuot, Patrick Levy, M. Carmen Martinez, Frédéric Gagnadoux, Ramaroson Andriantsitohaina

**Affiliations:** 1 INSERM U1063, Sopam, Angers University, F-49045, Angers, France; 2 Department of Respiratory Diseases, Angers University hospital, Angers, France; 3 INSERM U1042, HP2 laboratory, Joseph Fourier University, Grenoble, France; 4 Laboratoires du Sommeil et EFCR, A. Michallon University Hospital, Grenoble, France; Imperial College London, UNITED KINGDOM

## Abstract

**Background:**

Experimental models of intermittent hypoxia (IH) have been developed during the last decade to investigate the consequences of obstructive sleep apnea. IH is usually associated with detrimental metabolic and vascular outcomes. However, paradoxical protective effects have also been described depending of IH patterns and durations applied in studies. We evaluated the impact of short-term IH on vascular and metabolic function in a diet-induced model of metabolic syndrome (MS).

**Methods:**

Mice were fed either a standard diet or a high fat diet (HFD) for 8 weeks. During the final 14 days of each diet, animals were exposed to either IH (1 min cycle, FiO_2_ 5% for 30s, FiO_2_ 21% for 30s; 8 h/day) or intermittent air (FiO_2_ 21%). Ex-vivo vascular reactivity in response to acetylcholine was assessed in aorta rings by myography. Glucose, insulin and leptin levels were assessed, as well as serum lipid profile, hepatic mitochondrial activity and tissue nitric oxide (NO) release.

**Results:**

Mice fed with HFD developed moderate markers of dysmetabolism mimicking MS, including increased epididymal fat, dyslipidemia, hepatic steatosis and endothelial dysfunction. HFD decreased mitochondrial complex I, II and IV activities and increased lactate dehydrogenase (LDH) activity in liver. IH applied to HFD mice induced a major increase in insulin and leptin levels and prevented endothelial dysfunction by restoring NO production. IH also restored mitochondrial complex I and IV activities, moderated the increase in LDH activity and liver triglyceride accumulation in HFD mice.

**Conclusion:**

In a mouse model of MS, short-term IH increases insulin and leptin levels, restores endothelial function and mitochondrial activity and limits liver lipid accumulation.

## Introduction

Obesity is a rapidly growing problem that is reaching epidemic proportions worldwide, and is associated with an increased risk of cardiovascular morbidity and mortality [1, 2]. The clinical and metabolic consequences of obesity are usually grouped under the term of metabolic syndrome (MS), which is defined as a combination of abnormalities including central obesity, hypertriglyceridemia, low levels of HDL cholesterol, hypertension and hyperglycemia. In addition to cardiovascular comorbidities, obesity is also associated with non-alcoholic fatty liver disease (NAFLD), the hepatic expression of the MS [3]. Several clinical studies have demonstrated that NAFLD *per se* may constitute a cardiovascular risk factor independently of obesity and of MS components, as attested by the independent association between NAFLD and endothelial dysfunction [4, 5]. Furthermore, overweight and obesity also constitute the main risk factor for obstructive sleep apnea (OSA). Indeed, two-thirds of MS patients present moderate to severe OSA [6]. OSA is per se recognized as an independent risk factor for cardiovascular [7] and more recently liver diseases [8]. The frequent association of OSA and obesity in clinical setting makes it difficult to investigate their independent contribution to metabolic and vascular dysfunction.

To overcome those limitations, animal models of intermittent hypoxia (IH), mimicking the desaturations observed in OSA, have been developed over recent decades [9]. IH is thought to be a major component of OSA and to play an important role in hepatic, metabolic and vascular consequences of OSA, including impaired glucose and lipid metabolism [10, 11], increased risk of NAFLD [8] and cardiovascular morbidity [12].

Animal models of obesity and IH have been both associated with an increase in adipose tissue lipolysis and free fatty acids release [13–15]. Free fatty acids prevent tyrosine phosphorylation of the insulin receptor substrate, which decreases the ability of insulin to stimulate glucose transport, leading to insulin-resistance [16]. Fatty acid accumulation in the liver participates in the hepatic steatosis and toxicity. Furthermore, hepatic mitochondrial dysfunction leading to increased oxidative stress and altered free fatty acid oxidation have been described in obesity [17]. Subsequently insulin-resistance, dyslipidemia and oxidative stress are known to participate in the endothelial dysfunction and atherosclerosis.

In contrast, IH has also been proposed as a technique to improve physiological performance based on the phenomenon of adaptation to reduced oxygen, similar to mechanisms observed with ischemia-reperfusion preconditioning. IH has been shown to improve endothelial function in hypertension [18] and to limit infarct size [19]. The opposite outcomes observed in studies using IH can be influenced by the diversity and duration of IH patterns applied. Furthermore, the presence of underlying conditions such as age, or genotypic variation may represent important factors tilting the balance between an appropriate homeostatic response and decompensation [20].

The aim of this study was to test the impact of a short-term IH on vascular, metabolic and hepatic function in an animal model of MS.

## Methods

### Animals and protocol design

Ninety-six wild-type, 8-week-old male, C57BL/6J mice purchased from Janvier Laboratory (Paris, France) were used in this study. The study was conducted in accordance with the European Convention for the Protection of Vertebrate Animals used for Experimental and Other Scientific Purposes (Council of Europe, European Treaties ETS 123, Strasbourg, 18 March 1986), and the Guide for Care and Use of Laboratory Animals (NIH Publication No. 85–23, revised 1996) and was approved by the ethical committee for animal research of Grenoble. Forty-eight mice were fed with a standard diet (SD) (3.3 kcal/g) and 48 mice were fed with a high-fat diet (HFD) (SAFE, Augy, France) (42% of calories derived from fat, 15.2% from proteins and 42.7% from carbohydrates; 4.5 kcal/g) for 8 weeks. This HFD duration has been shown to induce early signs of obesity associated with endothelial dysfunction [21].

IH was performed as previously described [22]. A gas control delivery system was designed to regulate the flow of room air and nitrogen into customized cages housing the mice. During each period of IH, the FiO_2_ was reduced from 20 to 5.0% over a 30 s period and then rapidly reoxygenated to room air levels in the subsequent 30 s period. As previously described [22], mice placed in identical cages receiving intermittent air (IA) at similar flows were used as a control group. The IA control group allowed us to evaluate the specific impact of IH independently of noise and turbulences related to gas circulation. Animals were divided into four groups: (1) IA with SD (IA-SD), (2) IH with SD (IH-SD), (3) IA with HFD (IA-HFD) and (4) IH with HFD (IH-HFD). Mice on SD and on HFD were placed in the IH or IA chamber for the last two consecutive weeks of the experiment. The IH and IA states were induced for 8 hours during the light phase alternating with constant room air during the dark phase.

Mice were fasted for 8 hours before bleeding and sacrifice. Arterial blood (about 1 ml) was obtained by direct cardiac puncture under pentobarbital anaesthesia. The thoracic aorta was dissected and placed in modified Krebs-Henseleit-Hepes solution (KHS) for vascular reactivity analyses (n = 32) or immediately incubated with specific solutions for nitric oxide (NO) analysis (n = 32). Descending aorta and liver were surgically removed and immediately frozen at—80°C for subsequent analysis (n = 32). Adiposity was measured using the epididymal fat weight.

### Biochemical profiles

Serum samples were obtained from blood by centrifugation for 15 min at 950 *g* and room temperature. Insulin and leptin levels were measured using commercial Elisa kits (Millipore, Billerica, Massachusetts; Merck Millipore, Darmstadt, Germany). Fasting glucose, triglycerides (TG) and total cholesterol were measured with respective enzymatic kits from Thermo Scientific (Vantaa, Finland).

### Triglyceride content in the liver

Liver TG content was determined using the method described by Xu et al. [23]. Briefly, 100 mg of liver tissue was homogenized in 1 ml of ethanol followed by centrifugation at 15,000 *g* for 10 min, and TG levels were measured with a Sigma-Aldrich kit (Sigma-Aldrich, St Quentin Fallavier, France).

### Mitochondrial enzyme activities in liver homogenates

Liver homogenates were prepared as previously described and citrate synthase (CS), lactate dehydrogenase (LDH), complex I, II, III and IV activities were measured spectrophotometrically at 37°C by an adaptation of the method described by Malgat et al. [24] and in agreement with the Mitochondrial Diseases Group of the *Association Française contre les Myopathies*. LDH activity was determined by monitoring oxidation of NADH at 340 nm when incubated with pyruvate, as described previously [25]. CS activity was measured by monitoring the change in optical density of 5,5’-dithio-bis(2-nitrobenzoic acid) at 412 nm. NADH ubiquinone reductase activity (complex I) was determined by monitoring oxidation of NADH at 340 nm. Succinate ubiquinone reductase (complex II) was measured by monitoring reduction of 2,6-dichlorophenolindophenol at 600 nm. Ubiquinone cytochrome c reductase activity (complex III) was determined by monitoring reduction of cytochrome c at 550 nm. Cytochrome c oxidase (complex IV) activity was measured by monitoring oxidation of reduced cytochrome c at 550 nm. CS and LDH activities are expressed in nmol/min/mg protein. Other results are expressed as the ratio of enzyme activity and CS activity.

### Vascular reactivity

The thoracic aorta was dissected and placed in modified KHS, final composition in mmol/l: NaCl 130, KCl 4.7, MgSO_4_ 1.2, CaCl_2_ 2.5, HEPES 15, and glucose 5. Aortic rings (1.5–2 mm in length) were mounted on a wire myograph (Danish MyoTechnology, Aarhus, Denmark) filled with KHS, as described previously [26]. Arterial segments were stretched to a resting tension of 5 mN and allowed to equilibrate for 30 min. Endothelium-dependent vasodilatation in response to acetylcholine (ACh, 1 nM to 10 mM) was studied in aortas pre-contracted with the thromboxane A2 agonist (9,11-dideoxy-11α, 9α epoxymethanoprostaglandinF2α) U46619 at 80% of their maximal response.

### NO determination by electron paramagnetic resonance (EPR)

NO production was detected by the Fe^2+^ diethyldithiocarbamate (DETC; Sigma-Aldrich) spin trap technique, as previously described [27]. Aortas were dissected and incubated for NO production for 30 min in Krebs—Hepes buffer containing: BSA (20.5 g/l), CaCl_2_ (3 mM) and L-Arginine (0.8 mM) (Sigma-Aldrich). NaDETC (1.5 mM) and FeSO_4_.7H_2_O (1.5 mM) (Sigma-Aldrich) were dissolved separately under argon gas bubbling in 10 ml volumes of ice-cold Krebs—Hepes buffer and were then rapidly mixed to obtain a pale yellow-brown opalescent colloid Fe(DETC)_2_ solution (0.4 mM), which was used immediately. The colloid Fe(DETC)_2_ solution was added to vessels and incubated for 45 min at 37°C. Arteries were then immediately frozen in plastic tubes using liquid nitrogen. NO measurement was performed on a table-top x-band spectrometer Miniscope (Magnettech, MS200; Berlin, Germany). Signals were quantified by measuring the total amplitude, after correction for baseline. Values are expressed in arbitrary units/mg weight of dried tissue.

### Western Blotting

The expression level of target proteins was analysed by the Western blot technique. Frozen aortas were lysed separately in a buffer containing TRIS-HCl (10 mM), KCl (50 mM), EDTA (1 mM), 1% NONIDET P-40 and 3% Protease Inhibitor Cocktail (Sigma-Aldrich). Protein concentration was measured using the Protein Assay (Bio-Rad Laboratories, Hercules, CA) according to the manufacturer’s instructions. Equal amounts of protein (40 μg) were separated on 4–12% NuPAGE gel (Life Technologies, Carlsbad, CA) and transferred onto nitrocellulose membranes (Bio-Rad Laboratories, Hercules, CA). Membranes were probed overnight at 4°C with the following primary antibodies: anti-endothelial NO synthase (eNOS) (BD Biosciences, San Jose, CA), phospho-eNOS Ser 1177, phospho-eNOS Thr 495 (Cell Signaling, Beverly, MA), β-actin (Sigma-Aldrich). After washing three times with TBS-T, membranes were incubated with the appropriate horseradish peroxidase-conjugated secondary antibody (Pierce, Rockford, IL). Horseradish peroxidase reaction was detected using an enhanced chemiluminescent substrate, Super Signal West Dura Extended Duration (Pierce, Rockford, IL). Bands were scanned and their density quantified by means of BioRad GelDoc 2000 with reference to β-actin as loading control.

### Data analysis

Data are expressed as mean ± standard deviation; n represents the number of mice. Relaxation induced by ACh was expressed as a percentage of the maximal contraction. Graph-Pad Prism Software Version 5.0 (San Diego, CA) was used to calculate nonlinear regression. Differences between means were assessed by one-way analysis of variance followed by Tukey *post hoc* analysis or non-parametric tests (Kruskal—Wallis or Mann-Whitney U test) according to normality and variance homogeneity, except in vascular reactivity experiments where a two-way analysis of variance was used to compare concentration—response curves with agonists. Differences were considered significant when *p*<0.05.

## Results

### Food intake, body and organ weights, and lipid and glucose metabolism

Food intake was not affected in IH-SD mice as compared to IA-SD mice. In IA-HFD mice, daily food intake remained unchanged, but daily caloric intake was increased compared to IA-SD group. In IH-HFD mice, food intake was lower than in IA-HFD and IA-SD mice, and daily caloric intake was lower than in IA-HFD mice but similar to that of IA-SD and IH-SD mice (Table 1).

**Table 1:** Effects of short-term intermittent hypoxia (IH) and high-fat diet (HFD) on body weight, food intake, liver mass, epididymal fat pad, lipid metabolism, and liver triglyceride content in mice.

	IA-SD	IH-SD	IA-HFD	IH-HFD
N	24	24	24	24
Daily food intake				
g	3.3±0.8	3.4±0.9	3.1±0.8	2.5±0.6^#^ *
kcal	10.9±2.6	11.2±3.0	13.9±3.6*	11.2 ±2.7^#^
Weight gain, g	2.9±1.8	2.1±1.4	4.3±1.0* ^§^	2.0±1.3^#^
Liver weight, g	1.2±0.1	1.2±0.2	1.3±0.2	1.1±0.1
Liver weight/body weight, %	4.7±0.2	4.7±0.5	4.5±0.5	4.3±0.4
Epididymal fat weight, g	0.4±0.2	0.4±0.2	0.6±0.2* ^§^	0.6±0.2* ^§^
Epididymal fat weight/body weight, %	1.5±0,2	1.5±0.3	2.3±0.7* ^§^	2.1±0.7* ^§^
Fasting serum cholesterol, g/L	0.95±0.12	0.98±0.15	1.4±0.15* ^§^	1.4±0.16* ^§^
Fasting serum triglycerides g/L	0.61±0.14	0.63±0.15	0.60±0.17	0.59±0.11
Liver triglycerides mg/g	192±60	218±60	302±49*	218±245^#^

Values are expressed as mean ± standard deviation;

*p<0.05 vs IA-SD.

^#^ *p*<0.05 vs IA-HFD mice.

^§^ p<0.05 vs IH-SD. IA intermittent air, IH intermittent hypoxia, SD standard diet, HFD high fat diet

By the end of the 8-week protocol, IH-SD mice had similar weight gain as compared to IA-SD mice. Weight gain was significantly increased in IA-HFD group as compared to IA-SD group (Table 1). IH-HFD mice presented a lower weight gain compared to IA-HFD mice and a similar weight gain to that of IA-SD mice.

No differences in liver weight were observed between the four groups (Table 1). IH-SD had no impact on adipose tissue weight. HFD mice showed a significantly increased amount of epididymal fat compared to SD mice regardless of HI or IA treatment.

**Fig 1:**
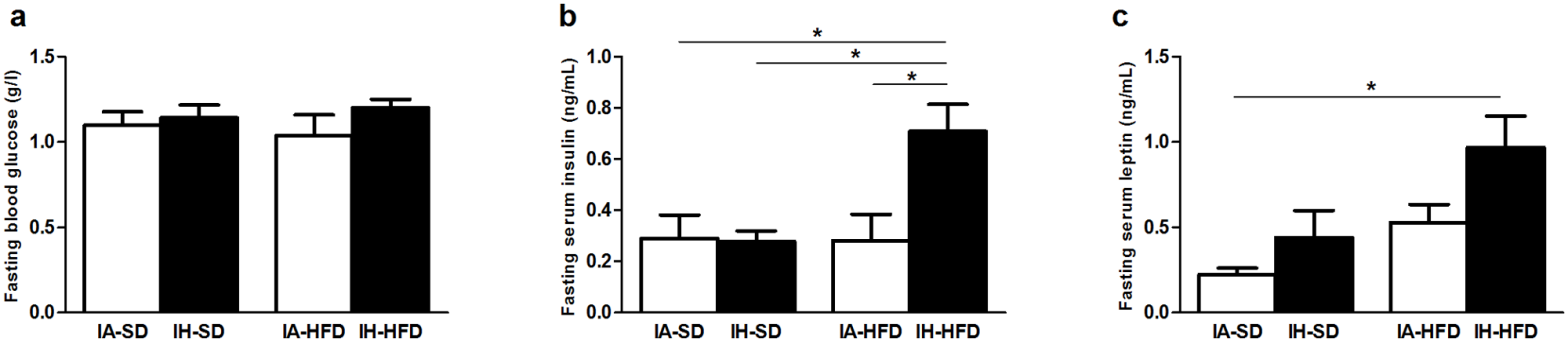
Plasma concentrations of (a) glucose, (b) insulin and (c) leptin, in intermittent air with standard diet (IA-SD), intermittent hypoxia with standard diet (IH-SD), intermittent air and high fat diet (IA-HFD) and intermittent hypoxia and high fat diet (IH-HFD) mice. Results are expressed as mean ± standard deviation and represent n = 6–8 mice, *p < 0.05

No changes in lipid metabolism were observed in IH-SD mice compared to IA-SD mice. HFD induced a significant increase in serum total cholesterol independently of IA or IH exposure. No differences in plasma TG levels were observed between the four groups (Table 1). Neither IH nor HFD alone induced any significant changes in fasting levels of glucose (Fig 1a) and insulin (Fig 1b). In contrast, the combined IH and HFD treatment induced a 2.5 fold increase in serum insulin as compared to IA-SD, the IH-SD and the IA-HFD group. IH and HFD alone induced a slight but non-significant increase in plasma leptin level. Interestingly, animals submitted to both IH and HFD showed a 4-fold increase in leptin level compared to IA-SD mice (Fig 1c).

### TG content in the liver

Liver TG content was not affected in IH-SD mice. IA-HFD caused an elevation in the liver TG content as compared to IA-SD mice, reflecting the presence of hepatic steatosis (Table 1). In contrast, the TG content observed in the IH-HFD group was not significantly different to that found in the IA-SD control group, suggesting that IH limited TG accumulation in the HFD model.

### Glycolytic and mitochondrial activity in liver homogenates

LDH activity was not affected in IH-SD mice. In contrast, an increased LDH activity was observed in liver homogenates from IA-HFD mice compared to IA-SD mice, suggesting an increase of glycolytic metabolism in IA-HFD mice (Table 2). The combination of HFD and IH did not affect LDH activity as compared to the IA-SD group.

**Table 2:** Effects of short-term intermittent hypoxia (IH) and high-fat diet (HFD) on glycolytic and mitochondrial activity in the liver homogenates.

	IA-SD	IH-SD	IA-HFD	IH-HFD
LDH	2428±575	2731±874	4142±531*	3123±1135
Citrate synthase	303.5±66.1	328.9±59.6	398.1±60.5	357.1±87.7
Complex I	0.48±0.09	0.43±0.07	0.37±0.07*	0.42±0.07
Complex II	0.68±0.12	0.72±0.18	0.42±0.18*	0.51±0.18*
Complex III	0.38±0.12	0.23±0.02*	0.30±0.15	0.21±0.09*
Complex IV	0.47±0.10	0.50±0.15	0.28±0.07*	0.34±0.14

CS and LDH activities are expressed in nmol/min/mg protein. Other results are the ratio of enzyme activity and CS activity. Values are expressed as mean ± standard deviation (n = 6)

*p<0.05 vs IA-SD mice.

No significant difference was observed for CS activity, suggesting no differences in liver mitochondrial mass between groups. IH-SD did not affect the activity of complexes I, II and IV, but significantly reduced the activity of complex III. IA-HFD mice displayed alterations in liver mitochondrial function, as demonstrated by decreased activity of respiratory chain complexes I, II and IV compared to the IA-SD group (Table 2). Complex III activity was also non-significantly decreased. Interestingly, in IH-HFD mice, complex I activity was restored to the levels observed in IA-SD mice. In IH-HFD mice, complex II was significantly reduced compared to IA-SD mice but was not statistically different from that of IA-HFD mice. Complex III activity was impaired in IH-HFD, suggesting a specific role of IH in decreasing complex III activity independently of dietary conditions. In IH-HFD mice, complex IV activity was restored toward that of IA-SD group, but was not significantly different from that of IA-HFD mice.

### Vascular reactivity

**Fig 2:**
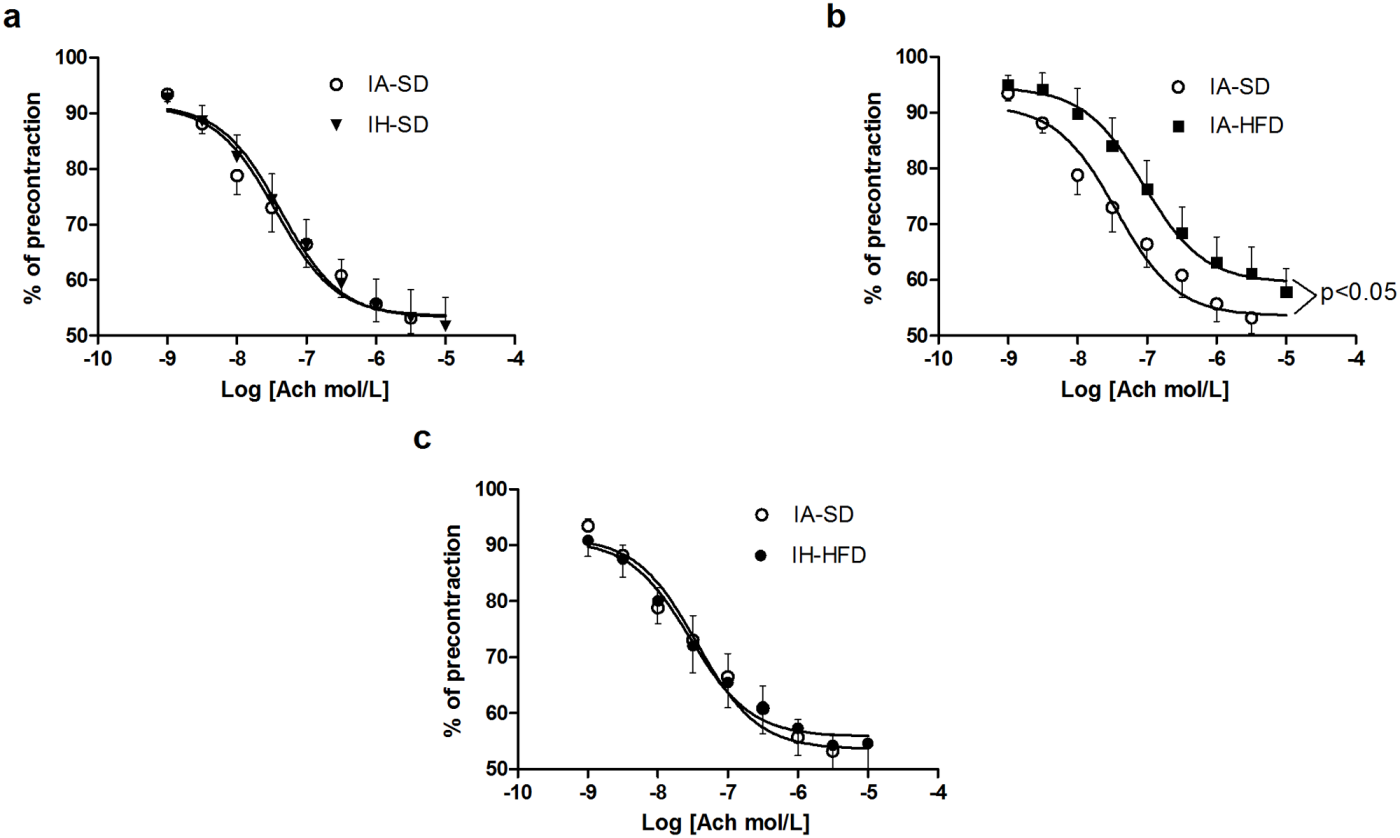
Concentration-response curves to acetylcholine (ACh) of aortic rings precontracted with the thromboxane agonist U46619 in the presence of functional endothelium. Vessels were isolated from intermittent air with standard diet (IA-SD), intermittent hypoxia with standard diet (IH-SD), intermittent air and high fat diet (IA-HFD) and intermittent hypoxia and high fat diet (IH-HFD) mice. Results are expressed as a percentage of relaxation of U46619-induced precontraction. (a) Vasodilator responses in the aorta from IA-SD and IH-SD mice. (b) Vasodilatation in vessels from IA-HFD mice compared to IA-SD mice. (c) Vasodilator responses in IH-HFD mice compared to IA-SD mice. Values are displayed as mean ± standard deviation and represent n = 8 mice. *p < 0.05 vs IH-SD. No significant differences were observed between IA-SD control, IH-SD and IH-HFD mice at any dose. An alteration of endothelial function was observed in aortic rings from IA-HFD compared with IA-SD mice.

IH associated with SD had no impact on endothelium-dependent relaxation of the aorta in response to cumulative addition of ACh as compared to IA-SD group (Fig 2a). Aortic rings from IA-HFD mice displayed reduced endothelium-dependent relaxation to ACh compared to IA-SD mice (P<0.05) (Fig 2b).

ACh-induced relaxation of aorta from IH-HFD mice was not affected as compared to IA-SD group (Fig 2c). The combination of the two treatments therefore protected against HFD-induced endothelial dysfunction.

### Tissue NO production

**Fig 3:**
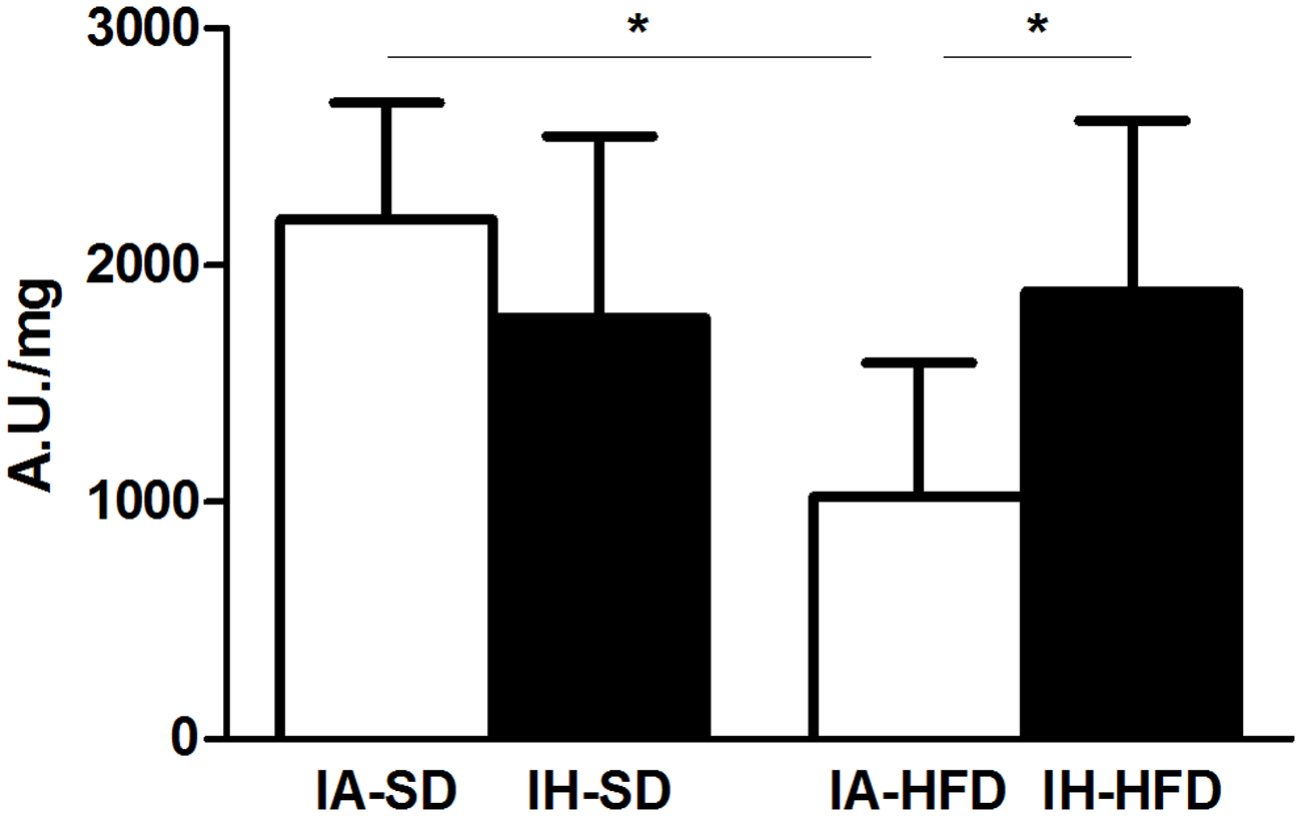
Nitric oxide (NO) production in aortas from intermittent air with standard diet (IA-SD), intermittent hypoxia with standard diet (IH-SD), intermittent air and high fat diet (IA-HFD) and intermittent hypoxia and high fat diet (IH-HFD) mice. Values are expressed in arbitrary units of amplitude per weight (mg) of dried tissue. Data are presented as mean ± standard deviation. And represent n = 5–7 mice *p<0.05.

IH-SD did not modify aortic NO production (Fig 3). In IA-HFD mice, NO production was decreased as compared to IA-SD group. Interestingly, the combination of IH and HFD reversed the reduced NO production so that the NO level was restored toward the IA-SD mice level.

**Fig 4:**
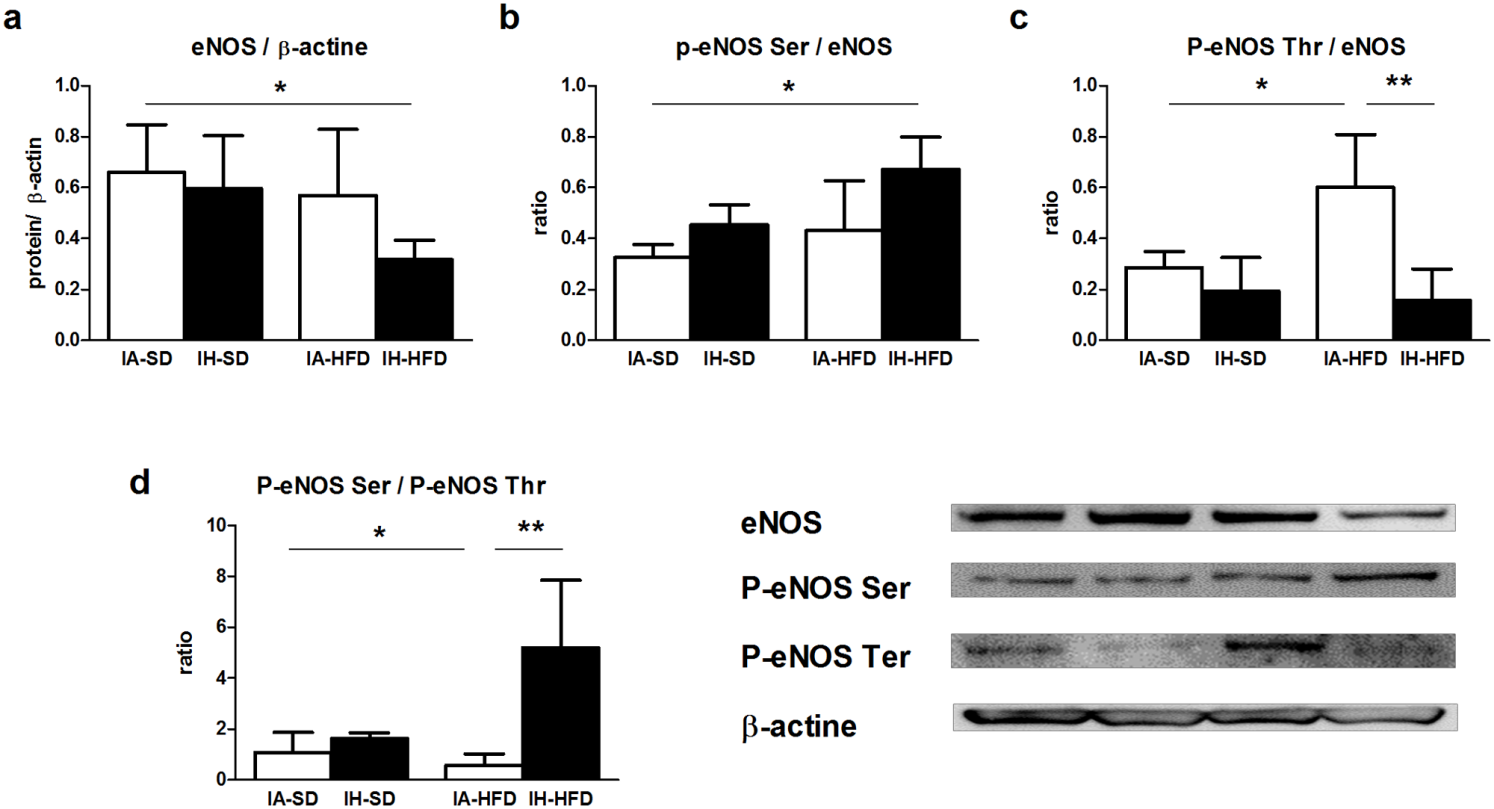
Western blotting performed in aortas from intermittent air with standard diet (IA-SD), intermittent hypoxia with standard diet (IH-SD), intermittent air and high fat diet (IA-HFD) and intermittent hypoxia and high fat diet (IH-HFD) mice using antibodies raised against (a) eNOS, (b) phospho-eNOS Ser 1177, (c) phospho-eNOS Thr 495. Immunoblots were quantified by densitometric analysis. Data are representative of 3–4 separate blots representing aorta lysates from 3–4 different mice. Densitometry values are expressed in arbitrary units (A.U.) as mean ± standard deviation. (d) The ratio of phosphorylation of eNOS at the inhibitor site (Thr 495) and activation site (Ser 1177) is expressed in arbitrary units (A.U.) as mean ± standard deviation and represent n = 3–4 mice *p< 0.05, **p <0.01.

In order to elucidate the molecular changes governing the HFD-induced reduction of NO release and restoration of NO production in the aorta from IH-HFD mice, we analysed the expression and activation of enzymes linked to the NO pathway by Western blotting. IH-SD did not modify either eNOS expression or phosphorylation of the activation and inhibitory sites of eNOS (Fig 4). IA-HFD did not change global eNOS expression (Fig 4a). However, in IA-HFD mice, eNOS phosphorylation at the activation site was not modified whereas that of the inhibitory site was markedly increased (Fig 4b and 4c). This results in a reduced ratio of P-eNOS Ser/P-eNOS Thr (Fig 4d) suggesting that IA-HFD decreased eNOS activity in accordance with the reduction in NO production (Fig 3). In the aorta from mice exposed to both IH and HFD (IH-HFD group), eNOS expression was significantly impaired (Fig 4a). However, eNOS phosphorylation was markedly increased at the activation site (Ser 1177) (Fig 4b) and decreased at the inhibition site (Fig 4c) which subsequently increased the ratio of P-eNOS Ser/P-eNOS Thr for IH-HFD mice (Fig 4d). Thus, the combination of both reduced eNOS expression and increased eNOS activity may explained the fact that IH restored the reduced aortic NO production induced by HFD. They also support the data that IH restored endothelial dysfunction upon HFD treatment.

Circulating malondialdehyde levels, as a measure of oxidative status, were not significantly different in the four groups of mice studied (data not shown). Thus, changes in oxidative stress might not participate in the restoration of aortic NO production and endothelial function upon HFD treatment under our IH experimental conditions.

## Discussion

Mice fed with HFD presented a dyslipidaemia, a hepatic steatosis and a marked endothelial dysfunction. The short-term IH protocol had no impact on metabolic, hepatic and vascular functions in mice fed with SD. Interestingly, mice exposed to both HFD and IH presented a significant increase in insulin and leptin levels together with a correction of endothelial function, a lower lipid accumulation, and a restored mitochondrial function.

We found that HFD induced early signs of hepatic steatosis with an increase in both hepatic TG content, and LDH activity as well as a hepatic mitochondrial dysfunction. LDH activity has been proposed to evaluate the glycolytic cell capacities [28]. Also, IA-HFD mice present an increase in the glycolytic metabolism that might compensate the alteration of the mitochondrial oxidative capacities. These data are in accordance with the central role of mitochondria in HFD induced NAFLD [29]. HFD has already been shown to decrease mitochondrial complex activity [21] and induce profound modifications in mitochondrial lipid composition [30]. These changes appear to play a key role in the resulting inhibition of fatty acid oxidation and mitochondrial oxidative-phosphorylation associated with increased mitochondrial reactive oxygen species (ROS) production and hepatic lipid accumulation.

We report here that short term IH has no effect on the liver of mice fed with SD but may have some beneficial effects on hepatic steatosis in the HFD fed group by restoring the hepatic TG content toward the level of IA-SD mice. IH also limited the glycolytic metabolism increase observed in the IA-HFD group as attested by the lower LDH activity in the IH-HFD group. IH applied to obese mice for 4 weeks to 6 months has been described as promoting steatohepatitis [15, 31]. In contrast, short IH stimulus has already been shown to decrease hepatic inflammation and hepatic oxidative stress in mice fed with HFD [32]. In the present HFD model, IH restored complex I activity and moderately increased complex II and IV activities, which may contribute to restore mitochondrial oxidative-phosphorylation and limit liver lipid accumulation. Previous studies have suggested some beneficial effects of IH on the mitochondrial function. Indeed, IH (cyclic hypoxia induced by 10% O_2_) has been shown to reduce the susceptibility to Ca^2+^-induced mitochondrial membrane depolarization in the brain, consistent with protection from injury [33]. Hypobaric long cycles of IH (4 hours) also attenuated the reduction of myocardial ATP content, mitochondrial ATP synthase activity, membrane potential and respiratory control ratios due to ischemia-reperfusion injury [34].

In contrast, IH impaired complex III activity regardless of diet. Complex III has been shown to be an important cellular O_2_ sensor that triggers pathways leading to induction of a variety of genes required for adaptation to hypoxic conditions [35]. In response to sustained hypoxia, complex III generates ROS and subsequently causes accumulation of the hypoxia-inducible factors (HIF) and initiate further gene expression [36]. HIF is described as a major transcription factor over expressed in response to sustain hypoxia. HIF activates molecular pathways involved in the adaptive response to hypoxia. Data reported in the literature on the effects of IH on HIF expression are controversial [37]. Under our experimental conditions, we did not find significant differences in the expression of HIF-1α both in the aorta and the liver and HIF-2α in the liver (S1 and S2 Figs). Further studies are needed to more clearly understand the consequences of reduced complex III activity by IH on both hepatic and vascular function and HIF expression and activity.

In the present study, besides early signs of MS and hepatic steatosis, HFD induced an endothelial dysfunction. As previously described, we found that HFD impaired NO production [38]. This effect was associated with reduced eNOS activity rather than eNOS expression. We found that two weeks of IH had no impact on endothelial function in mice fed with a normal diet but reversed the endothelial dysfunction observed with HFD. Discrepancies exist regarding the impact of IH on endothelial function. Underlying conditions such as obesity, genotypic variance, the duration and the severity of IH as well as the vessels investigated in the studies are important factors that impact adaptive and detrimental effects of IH on endothelial function. Two weeks IH has been shown to impair endothelial function in small cerebral and skeletal muscle vessels in rats [39, 40]. Six weeks of IH altered endothelial function in mice mesenteric arteries through a decrease in eNOS expression [41]. Despite clear aortic histological modifications with increased intima media thickness and inflammatory alterations [22], neither 14 days nor 35 days of IH modified aortic relaxation in response to ACh [42]. In a recent study, neither 6 weeks of IH, nor 6 weeks of a HFD applied alone did impact aortic vasodilation in mice but the endothelial function was impaired when IH and HFD were applied together [43]. In contrast, short-term IH was also shown to induce protective cardiovascular effects if applied for a short time (14 days) with a decrease in vascular oxidative stress and a reduction in endothelial cells apoptosis in mice [44].

Interestingly, in our model, IH reversed the endothelial dysfunction induced by HFD in the aorta. Short term IH has already been shown to improve endothelial function in hypertension [18] or ischemia/reperfusion lesions [45]. Our data extend the potential beneficial impact of short term IH in the context of obesity and MS. In our study, endothelial function and NO production were restored via increased eNOS activity, despite decreased eNOS expression. A specific role of increased insulin may contribute to the favourable eNOS phosphorylation state and NO production increase. A marked increase in insulin levels has been previously described in genetic or HFD-induced obesity mice exposed to IH [15, 32]. A direct impact of short-term IH on pancreatic beta-cells has been proposed with increased beta-cell proliferation [46] and increased insulin secretion [47]. Several studies have reported that insulin, in addition to its metabolic modulation, directly activates vascular endothelial Akt—eNOS signalling, leading to enhanced endogenous NO production [48, 49]. Binding of insulin to insulin endothelial receptor triggers its phosphorylation and activation via an intrinsic kinase activity, leading to tyrosine phosphorylation of the insulin receptor substrate (IRS) proteins [50]. Phosphorylation of the IRS activates different serine/threonine kinases such as Akt via PI3K signaling. [50]. In turn, Akt activates the eNOS by phosphorylation of serine residue 1177 [51, 52]. One can advance the hypothesis that the large increase in insulin levels observed in our HFD-IH group may contribute to prevent HFD-induced endothelial dysfunction.

We also report an increase in leptin levels in animals submitted to both HFD and IH. A similar additive impact of HFD and IH on leptin levels has recently been described in rats [53]. Furthermore, in leptin-deficient ob/ob mice submitted to long term IH, recombinant leptin infusion restored IH-induced vascular abnormalities toward normoxic wild type mice levels suggesting a beneficial impact of leptin on the vascular function [54]. One can speculate that the increased levels of leptin participate in the restored endothelial function observed in the IH-HFD group. Furthermore, the increase in leptin levels and the subsequent increase in metabolic rate might also have contributed to limit weight gain in the IH-HFD.

Some limitations should be taken into account when interpreting our findings. IH rodent models have been developed to mimic the effect of OSA-associated oxygen desaturation. However, as OSA is a chronic condition, 2 weeks of IH might be probably too short to be representative of the long term IH exposure experienced by OSA patients in clinical setting. Another concern is the absence of pCO_2_ control in our study. Clinical trials showed that pCO_2_ changes negligibly [55] or increases slightly [56] during the apneic episodes. For technical reasons, most IH exposure paradigms in rodents do not include CO_2_ supplementation and the resultant hyperventilation leads to hypocapnia. As eucapnic IH has been shown to have a more severe vascular impact [57] the hypocapnic model used in our study may have contributed to the paradoxical beneficial impact of IH in mice fed with HFD. We acknowledge that the present findings do not allow drawing any conclusion regarding long term effects of IH in patients with OSA. However, our results are in accordance with previous data showing a beneficial impact of IH on hypertension, endothelial function [18] and ischemia-reperfusion damages [19]. More recently, clinical trials showed that short term IH had positive impact in two components of the MS: diabetes [58] and hypertension [59]. Further studies are required to evaluate the clinical benefice of short term IH exposure in patients with MS.

In conclusion, we demonstrated in a mouse model of MS that short-term IH increases insulin and leptin levels, restores endothelial function and mitochondrial activity and limits liver lipid accumulation. These findings suggest that short term exposure to IH induces early adaptive and compensatory mechanisms and may represent an efficient way to modify obesity associated hepatic and vascular dysfunction without use of drugs.

## Supporting Information

S1 Fig Western blotting performed in aortas (A) and livers (b) from intermittent air with standard diet (IA-SD), intermittent hypoxia with standard diet (IH-SD), intermittent air and high fat diet (IA-HFD) and intermittent hypoxia and high fat diet (IH-HFD) mice using antibodies raised against HIF-1α. (TIF)

S2 Fig Western blotting performed in livers from intermittent air with standard diet (IA-SD), intermittent hypoxia with standard diet (IH-SD), intermittent air and high fat diet (IA-HFD) and intermittent hypoxia and high fat diet (IH-HFD) mice using antibodies raised against HIF-2α. (TIF)
