# Supplementary figures and images for: Vascular and Hepatic Impact of Short-Term Intermittent Hypoxia in a Mouse Model of Metabolic Syndrome
Source: PLoS One. 2015 May 18;10(5):e0124637. doi: 10.1371/journal.pone.0124637 (PMC4436258; doi:10.1371/journal.pone.0124637)

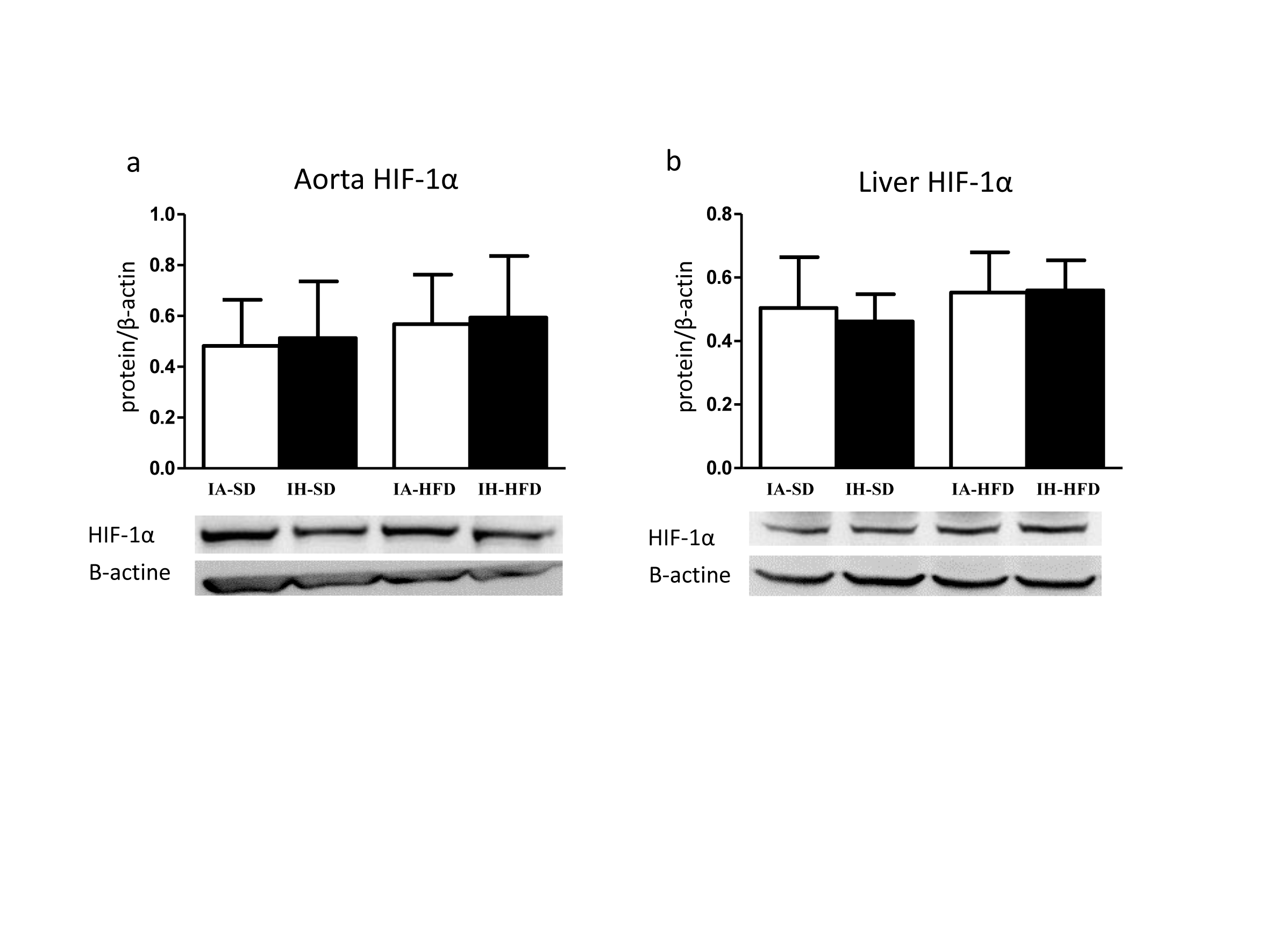

Supplement: S1 Fig — (TIF) [file pone.0124637.s001.tif]

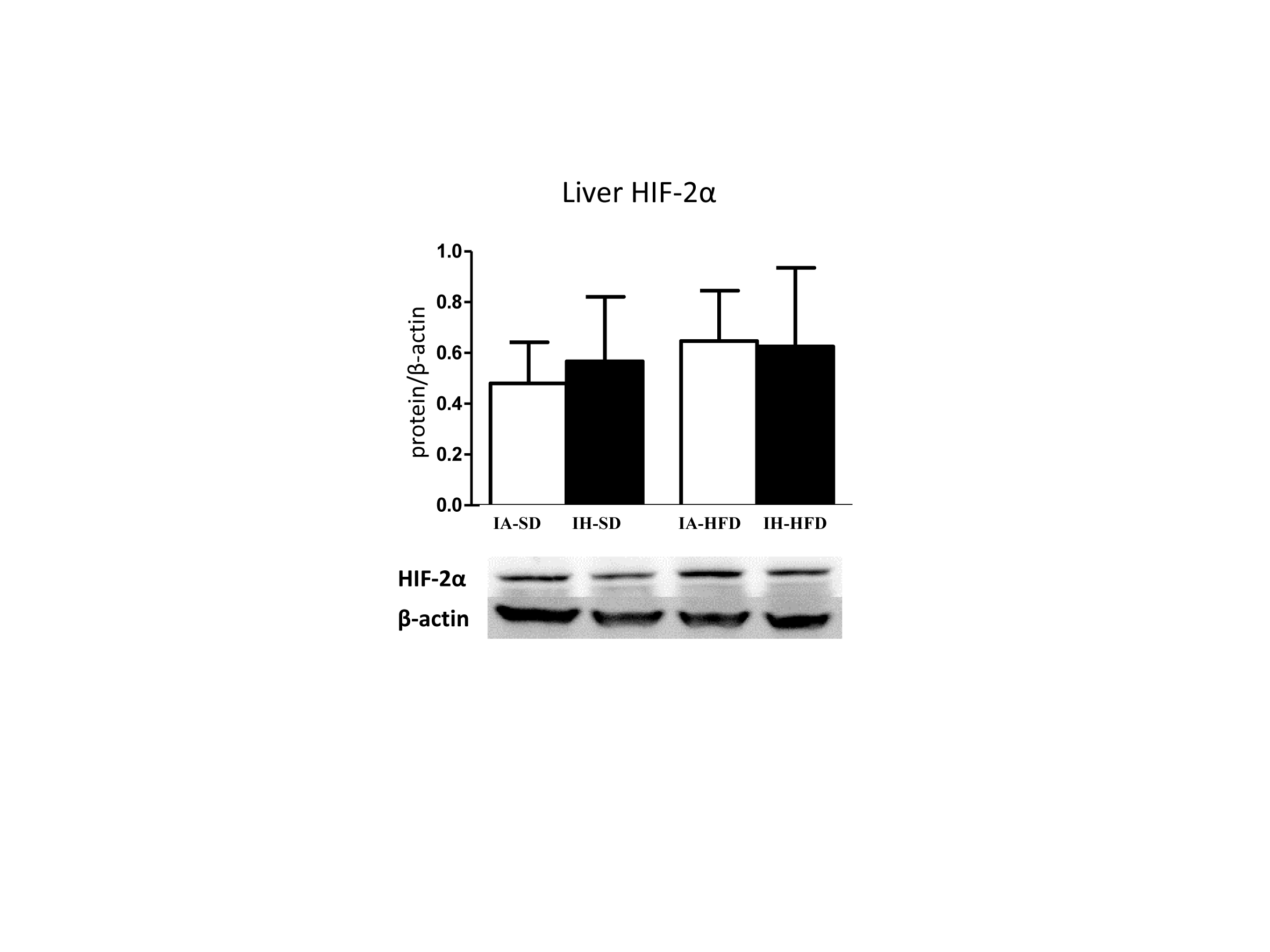

Supplement: S2 Fig — (TIF) [file pone.0124637.s002.tif]
